# Supplementary material for: HIVprotI: an integrated web based platform for prediction and design of HIV proteins inhibitors
Source: J Cheminform. 2018 Mar 9;10:12. doi: 10.1186/s13321-018-0266-y (PMC5845081; doi:10.1186/s13321-018-0266-y)
Supplement: Supplementary file 2 — Additional file 2. Source code of HIVProtI web server. [file 13321_2018_266_MOESM2_ESM.zip › HIVprotI_Source-code/hivprotI/weka-3-6-6/doc/weka/core/SelectedTag.html]

SelectedTag


---


|  |  |  |
| --- | --- | --- |
| **Overview** \| **Package** \| **Class** \| **Tree** \| **Deprecated** \| **Index** \| **Help** \| **Weka's home** | |  |
| **PREV CLASS**   **NEXT CLASS** | **FRAMES**    **NO FRAMES**     **All Classes** |  |
| SUMMARY: NESTED \| FIELD \| CONSTR \| METHOD | DETAIL: FIELD \| CONSTR \| METHOD |  |


---


## weka.core Class SelectedTag

```
java.lang.Object
  weka.core.SelectedTag
```

**All Implemented Interfaces:**: RevisionHandler

---

``` public class SelectedTag extends java.lang.Object implements RevisionHandler ```

Represents a selected value from a finite set of values, where each
value is a Tag (i.e. has some string associated with it). Primarily
used in schemes to select between alternative behaviours,
associating names with the alternative behaviours.

**Version:**
:   $Revision: 1.11 $

**Author:**
:   Len Trigg

---

**Constructor Summary**

`SelectedTag(int tagID,
Tag[] tags)`
  
          Creates a new `SelectedTag` instance.

`SelectedTag(java.lang.String tagText,
Tag[] tags)`
  
          Creates a new `SelectedTag` instance.


| **Method Summary** | |
| --- | --- |
| `boolean` | `equals(java.lang.Object o)`             Returns true if this SelectedTag equals another object |
| `java.lang.String` | `getRevision()`             Returns the revision string. |
| `Tag` | `getSelectedTag()`             Gets the selected Tag. |
| `Tag[]` | `getTags()`             Gets the set of all valid Tags. |
| `java.lang.String` | `toString()`             returns the selected tag in string representation |

**Methods inherited from class java.lang.Object**

`getClass, hashCode, notify, notifyAll, wait, wait, wait`

**Constructor Detail**

### SelectedTag

```
public SelectedTag(int tagID,
                   Tag[] tags)
```

:   Creates a new `SelectedTag` instance.

    **Parameters:**: `tagID` - the id of the selected tag.: `tags` - an array containing the possible valid Tags. **Throws:**: `java.lang.IllegalArgumentException` - if the selected tag isn't in the array of valid values or the IDs/IDStrs are not unique.

---


### SelectedTag

```
public SelectedTag(java.lang.String tagText,
                   Tag[] tags)
```

:   Creates a new `SelectedTag` instance.

    **Parameters:**: `tagText` - the text of the selected tag (case-insensitive).: `tags` - an array containing the possible valid Tags. **Throws:**: `java.lang.IllegalArgumentException` - if the selected tag isn't in the array of valid values.


**Method Detail**

### equals

```
public boolean equals(java.lang.Object o)
```

:   Returns true if this SelectedTag equals another object

    :   **Overrides:**: `equals` in class `java.lang.Object`
    :   **Parameters:**: `o` - the object to compare with **Returns:**: true if the tags and the selected tag are the same

---


### getSelectedTag

```
public Tag getSelectedTag()
```

:   Gets the selected Tag.

    :   **Returns:**: the selected Tag.

---


### getTags

```
public Tag[] getTags()
```

:   Gets the set of all valid Tags.

    :   **Returns:**: an array containing the valid Tags.

---


### toString

```
public java.lang.String toString()
```

:   returns the selected tag in string representation

    :   **Overrides:**: `toString` in class `java.lang.Object`
    :   **Returns:**: the selected tag as string

---


### getRevision

```
public java.lang.String getRevision()
```

:   Returns the revision string.

    :   **Specified by:**: `getRevision` in interface `RevisionHandler`
    :   **Returns:**: the revision


---


|  |  |  |
| --- | --- | --- |
| **Overview** \| **Package** \| **Class** \| **Tree** \| **Deprecated** \| **Index** \| **Help** \| **Weka's home** | |  |
| **PREV CLASS**   **NEXT CLASS** | **FRAMES**    **NO FRAMES**     **All Classes** |  |
| SUMMARY: NESTED \| FIELD \| CONSTR \| METHOD | DETAIL: FIELD \| CONSTR \| METHOD |  |


---
